# Reimagining nutrition education for pregnant adolescents in the face of climate change: a community approach

**DOI:** 10.1136/bmjnph-2023-000745

**Published:** 2024-04-24

**Authors:** Fleur de Meijer, Mary Kimanthi, Susan Cheruiyot, Alex Makau Muia, Donnah Goga, Soulthy Azamkhan Mohamed, Cecilia Njoga, Catherine Gathu, Felix Agoi, Nelson Nyamu, Jacob Shabani

**Affiliations:** 1 Family Medicine, The Aga Khan University, Nairobi, Kenya; 2 Population Health, The Aga Khan University, Nairobi, Kenya

**Keywords:** climate change, community health, community oriented primary care, nutrition education, food insecurity

## Abstract

**Objectives:**

To explore challenges with current nutrition education for teenage pregnant women in a drought-prone community in Kenya and to elicit the communities’ suggestions on how to best adapt it in the face of climate change.

**Design:**

Nine serial focus group discussions (four with adolescents, two with their parents, two with community health volunteers and one with healthcare workers) were conducted on a purposively selected study population in Kaloleni, Kilifi County, Kenya. Data collection took place between March and November 2022, with a total of 73 participants. An inductive approach was used, and interpretive thematic coding was done as the primary analytic strategy to allow themes derived from participants’ reflections.

**Results:**

First, participants reported that unpredictable rainfall patterns had affected nutrition intake and variety due to reduced yield from farmland, diseases in livestock and insufficient income. Second, participants reported barriers to accessing nutrition education, as it was mainly given in clinics and not targeted at adolescents or men. Third, they experienced challenges in applying nutrition education in daily life due to a mismatch between available foods and cultural practices. Recommendations for the future encompassed equipping individuals with practical cooking skills tailored to available nutrients, initiatives aimed at water conservation and addressing animal health concerns, enhancing accessibility through community-based training programmes and fostering collaborative efforts to ensure the provision of essential nutrients.

**Conclusion:**

Food choices in Kilifi County are getting more limited due to unpredicted rainfall patterns. Therefore, a reorientation of nutrition education is needed in order to build resilience in the community. Strengthening community action, including developing skills to increase long-term local support, would be needed to ensure the adequate nutrition status of vulnerable groups like pregnant adolescent women.

**WHAT IS ALREADY KNOWN ON THIS TOPIC**

- Climate change worsens undernutrition in vulnerable groups like pregnant adolescents and their children.

**WHAT THIS STUDY ADDS**

- The study demonstrates that existing nutrition education offered to pregnant adolescents in Kilifi County lacks adaptability to drought effects and food scarcity.

**HOW THIS STUDY MIGHT AFFECT RESEARCH, PRACTICE, OR POLICY**

- Culturally sensitive, practical nutrition education is crucial for underserved communities to improve their daily nutrition practices.

## Background

Climate change has disproportionately affected low-income and middle-income countries (LMICs), with unpredictable rainfall patterns and reduced food availability as a result. This especially threatens infants and adolescent pregnant women, who are most at risk for undernutrition. Pregnancy in adolescents is associated with an up to 50% higher chance of neonatal and maternal complications.^1^ In addition, children born to adolescent mothers are prone to undernutrition and later neurodevelopmental delays.^2 3^ Nutrition promotion and education programmes must consider these impacts and adapt to local communities' needs to enhance resilience to the recent climatic conditions.

The nutritional challenges faced in pregnancy are compounded in adolescent girls due to biological, psychological, social and cultural factors. Adolescence is characterised by a growth spurt, brain and emotional development, the appearance of secondary sexual characteristics and the redistribution of muscle and fat.^4 5^ These changes necessitate increased caloric intake to meet the body’s metabolic demands. Competing caloric demands between the mother and the growing fetus may result in nutrient deficiencies in the pregnant adolescent.^6^ After birth, inadequate breast milk production may push mothers towards an early introduction of complementary feeding. These poor infant feeding practices have been linked to childhood undernutrition and diarrhoeal diseases.^6 7^ In the long term, these result in stunting children and impaired mental growth and cognitive function.^5 7^

Psychologically, teenagers are inadequately equipped to deal with the demands of starting family and partner relationships.^8^ Financial insecurity, early marriage and dependence on parents or caregivers are factors shown to affect a young person’s capacity to implement good nutritional choices during pregnancy and the postpartum period.^9^ Pregnant adolescents encounter difficulties accessing appropriate maternal-related health services at the service delivery level due to stigma and the lack of youth-friendly services tailored for them.^10–14^

**Figure 1:**
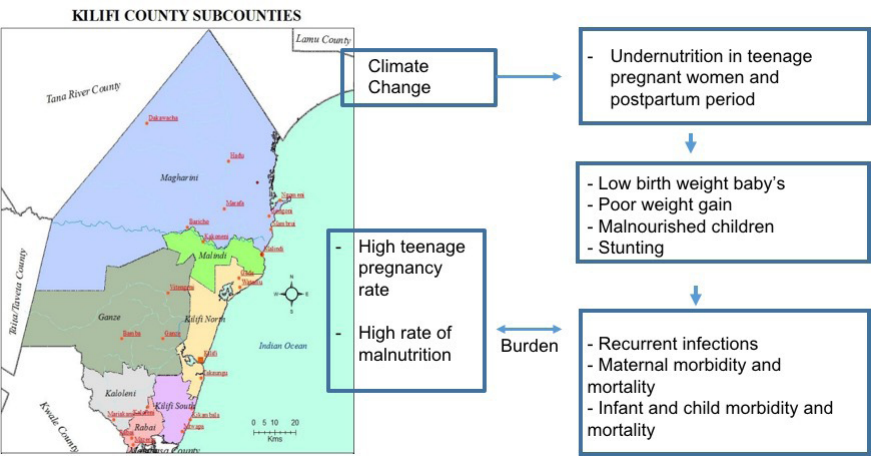
Potential effects of climate change on maternal and child morbidity in Kilifi.

Recent environmental changes have posed an extra challenge to effectively educating adolescent women on how to feed themselves and their newborn children. Unpredictable rainfall patterns in Kenya in 2021 and 2022 have resulted in poor crop yields and low livestock milk production.^15^ In 2021, Kilifi County in particular experienced a failure of the long rains and later flash floods that ruined the few crops in the fields. As a result, documented household milk production decreased from a high of 8–2 L/household/day.^15 16^ With the current teenage pregnancy burden within Kilifi County estimated at as high as 13%,^17^ neonatal mortality at 24% and 20% of children under 5 years being underweight (much higher than the national average of 10%), increasingly harsh climatic conditions will only further worsen the nutritional status of this high-risk population and cause a ripple effect on maternal and neonatal morbidity and mortality (see figure 1).^17–19^

Providing nutrition services targeting pregnant adolescents presents a unique opportunity for addressing these challenges.^20^ Nutrition education refers to learning experiences designed to facilitate the voluntary adoption of eating and other nutrition-related behaviours conducive to health and well-being.^20 21^ Strategies to adapt nutrition education to reach adolescents in the face of climate change have not been explored much. This study aims to understand different stakeholders’ challenges on how climatic change influences nutrition, experiences with current nutrition education for adolescent pregnant women and suggestions for adaptation of nutrition education programmes.

## Methods

### Study design and context

This study adopted a qualitative approach by conducting serial focus group discussions (FGDs) using a structured questionnaire. This strategy was chosen to better understand the nutritional difficulties over time and in different periods of rainfall (March–April and October–November).

Family Medicine residents from Aga Khan University in Kenya undertook the study as part of the department’s Community Oriented Primary Care programme, which aims to involve the community in finding context-appropriate solutions for local health problems. Kilifi is one of 47 counties in Kenya, located in the southeastern part, with a population of 1.5 million people. Almost half of the county’s working population is in casual employment, relying on farming or small-scale businesses for sustenance.^18^ Tsangatsini dispensary is a small health facility with a labour ward that acts as the primary referral facility for the community health units. Currently, nutrition education is done by community health volunteers (CHVs) at the dispensary and is given to all pregnant women. There is no specific education given to adolescent pregnant women. Supplements (iron) are supplied by the government.

### Study participants

The study participants included adolescent mothers and mothers-to-be, their parents and spouses, CHVs and healthcare workers (HCWs) attached to the Tsangatsini dispensary. The list of adolescent study participants was generated from facility records and a snowball sampling technique. Initial contact was made through CHVs during home visits or via a phone call where the purpose of the study was communicated. The researchers (MK and SC) approached CHVs and HCWs directly at the dispensary to explain the study. The second series of FGDs was held in October and November 2022, and the same participants were invited.

### Inclusion criteria

*Inclusion criteria* were as follows: adolescent girls and boys aged 15–19 years who were expecting or had children within 1 year of study (March 2021–March 2022); parents of adolescent girls or boys aged 15–19 years who had given birth or were pregnant within 1 year of study (March 2021–March 2022); CHVs working in the Tsangatsini area and HCWs domiciled at the dispensary.

### Ethical consideration

Ethical clearance to conduct the study was obtained from Aga Khan University (2021-IERC81/V5). The study was approved by the National Commission for Science, Technology and Innovation (Nacosti/P/22/15865). Before every FGD, written consent was sought from all the participants. The pregnant adolescents or adolescent mothers (15–19 years of age) were considered emancipated and therefore eligible to give informed consent. The majority managed to sign, while a thumbpad was available for those who could not. Participants were reimbursed for transport and provided a snack after the FGD.

### Data collection

FGD participants were separated into small groups (between five and nine participants) according to gender and age to ensure everyone was comfortable sharing their experiences. Mixed gender groups existed for CHVs and HCWs, as they were thought to be less influenced by gender differences. Two facilitators (MK, SC, FA, SA, CN, DG and AM) conducted each FGD, lasting between 60 and 90 min, at a suitable and quiet location (the church, under trees or inside the hospital meeting room). Adolescent groups were conducted by same-gender facilitators. The FGD facilitators were fluent in English and Kiswahili and received training from two experienced qualitative researchers to familiarise them with the objectives, topic guides and questioning techniques. The FGD questions were pilot tested with two community members, who did not participate in the FGDs, after which words were chosen that better resonated with the community. FGD questions centred on the participants’ personal and observed challenges to nutrition, their views on current health promotion and education and suggestions for the future (see online supplemental appendices 1 and 2). Interview guides tailor-made for each group were used to conduct the FGDs. Kiswahili, the language commonly spoken by the locals, was used in the discussions with the provision of translation from Giriama during the FGD. Participants were allocated a number at the start of the FGDs for deidentification. The discussions were audio-recorded, and the recordings were immediately submitted for transcription by an independent party. All the recordings were transcribed verbatim and translated into English. The transcripts were then sent to the primary investigators for data analysis.

10.1136/bmjnph-2023-000745.supp1 Supplementary data

### Data analysis

Excel software was used to store and manage the dataset. The Consolidated Criteria for Reporting Qualitative Research were followed for reporting the results of this study (see online supplemental appendix 3).^22^ An inductive approach was taken to analyse participants’ reflections, and interpretive thematic coding was done as the primary analytic strategy.^23^ Two female investigators (MK and SC) first read and reread the transcripts independently in order to extract codes emerging from the data in March and April 2022. This initial coding informed the second series of FGDs in October–November 2022 and deeper exploration of topics was done. Two different investigators (DG, female, and AM, male) then analysed the transcripts in October–November 2022 in order to minimise bias and to allow for any new emergent codes or themes. Saturation was concluded when no new information was acquired during the interviews and no new codes occurred in the data. MK and SC then compared the codes from the different transcripts and grouped codes with similar messages into categories. The second level of analysis involved a third researcher (FM, female) to review the transcripts and evaluate these initial codes and the appropriateness of categories across the different periods. In case of any disagreement, suitability was discussed with the other two researchers until consensus was reached and relevant themes across all stakeholders could be identified. A written record was kept by FM detailing what was decided during the coding process and why. The researchers ensured that theme names matched the language the stakeholders used. Subthemes were also identified and collapsed when appropriate. The second series of FGDs was also suitable to do members checking and validating information from the FGDs in March 2022.

## Results

### Participants

FGDs were held with a total of 73 participants. In March 2022, 24 adolescents (15–19 years of age), 18 CHVs, 10 healthcare workers and 14 parents joined the FGDs. Only 6 of the 24 adolescent girls were still in school, and 15 already had another child. Parents had an average of six children. The healthcare workers had worked for an average of 9.6 years in their current position. In November 2022, a second series of FGDs with the same participants was done, whereby five participants could not make it to the second round. One FGD with seven male adolescents was added, as no male adolescents had participated in March 2022. A summary of the demographic characteristics is shown in table 1.

**Table 1:** Demographic characteristics of study participants

Group	Periods FGD conducted	Number of participants	Mean age	Gender distribution	
				Female	Male
Adolescents 1	Both	8	18.6	8	0
Adolescents 2	Both	9	18.3	9	0
Adolescents 3	Both	7	18	7	0
Adolescents 4	Oct/Nov 2022	7	20	0	7
CHVs 1	Both	9	39.7	8	1
CHVs 2	Both	9	43.7	7	3
Healthcare workers	Both	10	29.2	4	5
Parents 1	Both	8	45.5	8	0
Parents 2	Both	6	40.7	6	0
Total		73	18.7	57	16

Note: In October–November 2022, five female participants (two adolescents, one CHV and two parents) did not participate in the second series of FGDs.

The healthcare workers consisted of three nurses, three community extension workers, a data clerk, a nutritionist and two health records and information officers.

CHVs, community health volunteers; FGD, focus group discussion.

### Qualitative evaluation of themes

**Figure 2:**
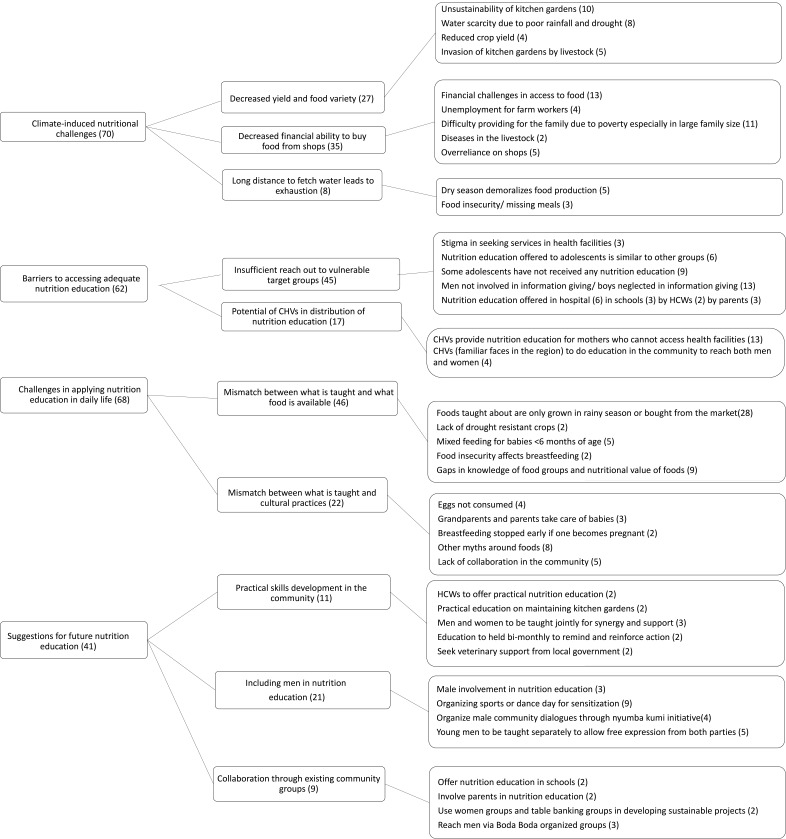
Coding tree focus group discussions.

Four themes emerged from the FGDs: (1) climate-induced nutritional challenges; (2) barriers to accessing adequate nutrition education; (3) challenges in applying nutrition education in daily life and (4) suggestions for future nutrition education. Themes and subthemes are described below in detail, with narratives from the various FGDs with each quote labelled. The coding tree used to arrive at subthemes and themes is provided in figure 2.

### Climate-induced nutritional challenges

All stakeholders reported increased difficulty adhering to a balanced diet due to changed weather patterns in the region.

#### Decreased yield and food variety

Unpredicted rainfall patterns in the last three consecutive years have contributed to reduced yield from farmlands. Participants reported that foods that were previously available are not available anymore as few vegetables thrive in periods of drought and proteins can now only be bought from stores. Many community members have kitchen gardens attached to their houses, but consecutive dry seasons have led to poor water access, resulting in the vegetables drying up and dying.

The easiest food to find here is Ugali, green vegetables when there’s rains… but as now we are in the dry season, you’ll find Sukuma…. but the other vegetables you have to look for them far… (adol1-2)

we can plant vegetables, fruits but they’re not there because we have a lot of sun and it has been there for a long time (parents1-4)

A prolonged drought has had an indirect impact on food production as lack of rainfall has reduced vegetation and grazing fields for animals, leaving them without an alternative food source. Participants report how goats have become a menace to farmers as they eat the available crops.

when the goats pass by, they eat them [vegetable gardens] so now they dried up (adol3-6)

#### Decreased financial ability to buy food from shops

Prolonged drought halts farming jobs, causing income loss and limiting access to the market and supplies.

It’s been a dry season; we have no work. If you get today, tomorrow you don't have. If you eat today, tomorrow you don't. (parents1-6)

This is problematic as essential proteins found in beans and lentils need to be bought from local markets. Also, there is an increased need to purchase milk from shops as illnesses in animals have caused insufficient livestock milk production. CHVs and parents also report that animal diseases have reduced their income from livestock.

there’s one we call “kidere” of which it affects mostly the chicks which have grown and you can’t sell and get profit. And that one we don’t have medication (parents2-7)

… the dry season has affected them until they died [goats] (adol3-7)

#### Exhaustion due to long distances for water

The continuous drought has caused nearby water pans and boreholes to dry up. Female community members report they often do not have the energy to work on the farmland, as they have to walk far to fetch water while having to look after the children too.

You can be overwhelmed on the journey (for water) if you have like almost 10 children, they'll line up crying to you, you'll also sit with them and start crying (adol3-7)

In the dry seasons, we do sprinkle the kitchen gardens. And for you to get this sprinkling water you have to buy it from the boreholes so that you sprinkle it. Now it’ll get to a point you’ll not manage. (chv2-7)

### Barriers to accessing adequate nutrition education

Access to current nutritional education and promotion services in the community was suboptimal due to several reasons.

#### Insufficient reach out to vulnerable target groups

The training provided primarily consists of theoretical sessions conducted at the Tsangatsini dispensary. However, female adolescents expressed apprehension about visiting the dispensary due to the stigma they may face from the community. This stigma is attributed to the fact that their age group is typically associated with visits to the clinic for pregnancy-related or HIV-related concerns. Unfortunately, the prevailing paternalistic culture perpetuates this fear and discourages open discussions about health among men in the adolescent group. Consequently, men in the community are not actively engaged in addressing health-related family issues, contributing to a significant information and support gap in the population’s overall well-being.

the (older) women take charge in matters on health and wellness. We are not benefiting from the discussions held at the health facility (adol4-2).

…nutrition is a family issue, but you'll find most times this information is being given to mothers only ….because the man didn't hear you saying it, they'll not take it seriously (chv1-6)

Parents and CHVs reported that there used to be home science classes preparing adolescents to cook varied meals. Adolescents’ nutrition knowledge and ability to create balanced meals have decreased with the cessation of these classes.

there was home science classes which were preparing these students in way that they won't struggle when they go back home to cook…but now, young people are preferring buying readymade food than preparing it themselves (chv1-6)

### Potential of CHVs in providing nutrition education

CHVs were mentioned as a source of nutrition knowledge in the community through home visits. Althoug CHVs do visit homes, nutrition education is not specified for adolescents and is often not followed up.

here's no special education given in regards to nutrition that is given to the young mothers. (chv1-9)

### Challenges in applying nutrition education in daily life

#### Mismatch between what is taught and what food is available

According to HCWs and CHVs, there is a lack of knowledge on sources of proteins from chicken and other food groups resulting in children being fed the same type of meal for breakfast, lunch and evening.

Mostly they give porridge, thereby it’s only one variety of food. They don't give fruits or vegetables or the meat. (hcw-2)

However, according to parents and adolescents, it was mostly drought that hindered them from applying what they knew.

foods that are found here, most times are porridge, black tea, and then foods they continue to eat is still Ugali because the food that they are taught here can’t be found (parents2-1)

If there is no water how will you cook? Can you really cook flour without water? (adol1-2)

#### Mismatch between what is taught and cultural practices

Cultural practices also hinder adolescents from applying what they know. For example, while many households rear chickens, women reported that their husbands do not allow them to use the eggs for cooking. Also, per cultural norms, a mother ceases breastfeeding upon learning about a new pregnancy, leading to a dependency on shop-bought milk when it is unavailable from goats.

They lay eggs but you're not allowed to touch the eggs (adoles1-3)

### Suggestions for future nutrition education

Stakeholders made several suggestions to adapt nutrition education in the community.

#### Practical skills development in the community

Adolescents, parents and CHVs suggested being educated on animal disease treatment as they depend highly on their livestock for income and milk. If medicines are needed, these would have to be provided as well.

So, if we could get help with that medication for the eye disease, you'll come to find that people will have chicken (chv 2-2)

Furthermore, they suggested that they be trained on techniques to preserve the little water available through storage or to be educated on planting drought-resistant crops. Moreover, they propose practical education based on available foods and which ones to prioritise rather than theoretical education.

let us know how to reserve that water to be of importance to our lives (adol2-9).

#### Including men in nutrition education

Although some CHVs suggested gender-specific training, most participants advocated for combined education forums for both men and women in the community to create a culture of synergy and support.

The men need to be taught about the importance of taking a lead in the health of their families as this will lead to better health for the whole (adol 4-5)

#### Collaboration through existing community groups

Parents and CHVs suggested more collaboration in supporting adolescent girls, as they may feel overwhelmed having to combine caring for their children with keeping kitchen gardens and fetching water.

Will I grow that or watch the baby? She finds it as an added burden. That is why my fellow parents are saying they should be also involved in the training. (parents 2-10)

Other suggestions were to work more actively with existing community groups. For example, training active women-saving groups or youth groups on cooking so they can subsequently mentor their peers. Also, active male boda boda (motorcycle operator) groups could be used to pass nutritional information to the rest of the young men in the community.

## Discussion

This study used qualitative data from serial FGDs with varied community stakeholders to evaluate nutritional education for teenage pregnant women in a drought-prone community in Kenya. Our findings demonstrate that current nutrition education is inadequately adapted to the effects of drought and ensuring sufficient balanced and nutritious food among the vulnerable population. Participants reported nutritional difficulties due to prolonged periods of drought, insufficient access to current nutrition education and difficulty translating what is taught into practice due to the unavailability of food and a mismatch with cultural practices.

### Skill development and collaborative support to improve access to food

Access to food in our study was affected both directly and indirectly by climate change: prolonged droughts and unpredicted rainfall had affected production from kitchen gardens and livestock directly, reducing overall calorie consumption as well as consumption of healthy foods, such as vegetables, fruits and animal-source foods. In Kenya, reduced precipitation in the southern areas, including Kilifi County, has negatively impacted the agricultural sector, which relies heavily on rain-fed food production.^19 24^ Reduced rainfall also affects crop production indirectly by reducing pasture, which forces livestock and sometimes even wild animals from local nature reserves, to feed on the kitchen gardens.^25 26^ Access to food was also indirectly affected due to livestock diseases and the loss of income generated from meat and dairy products. Climate change is known to make livestock susceptible to diseases as they suffer from heat stress and altered disease-causing pathogens.^27^ The implications of these consequences are significant, particularly in areas already vulnerable to food insecurity, as climate projections for Kenya show a rise between 1 and 4°C in average annual temperature in the decades to come.^21^

Nutrition education can play a crucial role in facilitating skill development in the community and mobilising resources in order to improve access to food. As suggested by the community, future efforts should focus on teaching young adolescents practical skills for farming, reserving water and treating livestock diseases. A recent study done among resource-poor Giriama farmers in South East Kenya showed that water conservation structures such as retention ditches and Zai-pits (small basins in which the seeds are planted) were well accepted by the community.^28^ Challenges, such as the high cost of labour for the establishment of such structures, could be faced by local support from the village leader. In many African countries, trust in community leaders is much higher than in elected government officials or donor organisations, which potentially facilitates effective leadership and establishing collaboration in the community to put up water structures or share essential nutrients.^29 30^ Support from the agricultural ministry through agricultural extension officers would be helpful to educate the community on treating livestock diseases and to provide pesticides and fertilisers.

### Improving access and acceptability of nutrition education

Another key finding from our study is that both female and male adolescents experienced insufficient access to current nutrition education. Providing nutrition education close to the community would be beneficial for women in our study who reported fear of stigmatisation in clinics and a lack of energy to walk long distances due to water scarcity. As climate change is known to not only affect women’s energy levels but also to complicate women’s time allocation to livelihoods and caregiving, it would be most efficient when nutrition education saves time and encompasses cooking classes, as suggested by the community.^29 30^ Also, men in our study felt excluded from current nutrition education. This is remarkable, as several studies have shown that male-headed households are more likely to adapt to climate change as they have more decision-making capacity.^31^ Inclusion of men is therefore crucial in order to curb food insecurities.

**Figure 3:**
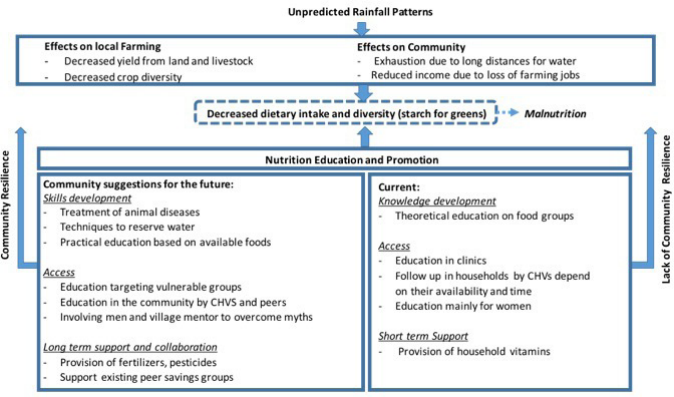
A proposed theoretical framework for building community resilience against undernutrition in the face of climate change.

Not only gender but also age and culture are important factors to consider in the provision of nutrition education.^32 33^ For example, adolescents in our study were reported to have a ‘purchase’ diet dominated by bought and processed foods, often containing much sugar. To bridge the gap between knowledge and behaviour change, future nutrition education efforts should focus on providing practical, actionable advice that individuals can easily incorporate into their daily lives.^34 35^ Also, blendingindigenous and scientific knowledge and involving the community is essential.^32^ Previous efforts to advise the community on mixing maize with other crops, for example, cassava, millet and/or sorghum and cowpeas failed, as these suggestions were made without the involvement of the community.^31 32 36^ Based on the findings above, we developed a framework that gave insight into the communities’ current strategies for nutrition education and suggested future actions that would make nutrition education more resilient against climatic changes (see figure 3).

### Study limitations

This study is limited to the Kilifi context, possibly affecting generalisation; nevertheless, it gives deep insight into the importance of seeking local solutions through a collaborative multistakeholder community process. The study’s findings can be extrapolated to similar communities in arid and semiarid areas. Future research should examine context-specific factors determining nutrition education in similar communities and generate community-specific solutions. Most of the study participants were female, highlighting the need for male involvement in healthcare decisions and interventions. Nevertheless, the large number of FGDs and the recruitment of different community stakeholders ensured the validity of the study findings. Further studies should be done to identify solutions for encouraging male involvement.

## Conclusion

Climate change exacerbates the existing malnutrition problem among vulnerable groups, such as teenage pregnant women, in Kenya. Our findings demonstrate that current nutrition education is inadequately adapted to the effects of drought and ensuring sufficient balanced and nutritious food.

## Recommendations

Based on the findings of our study, we suggest several areas for future nutrition education in Kilifi County. First, efforts should focus on developing strategies to promote climate-resilient food production and access to nutritious foods. Second, efforts should be made to expand access to nutrition education for both adolescent women and men. Third, nutrition education should focus on providing practical, actionable advice that individuals can easily incorporate into their daily lives.

## Data Availability

Data are available upon reasonable request.

## References

[1] Organisation WH . Adolescent Pregnancy Fact Sheet, Available: https://apps.who.int/iris/bitstream/handle/10665/112320/WHO_RHR_14.08_eng.pdf

[2] Thame M , Wilks R , Matadial L , et al . A comparative study of pregnancy outcome in teenage girls and mature women. West Indian Med J 1999;48:69–72. 10492605

[3] Wemakor A , Garti H , Azongo T , et al . Young maternal age is a risk factor for child Undernutrition in tamale metropolis, Ghana. BMC Res Notes 2018;11:877. 10.1186/s13104-018-3980-7 30526641 PMC6288872

[4] Norris SA , Frongillo EA , Black MM , et al . Nutrition in adolescent growth and development. Lancet 2022;399:172–84. 10.1016/S0140-6736(21)01590-7 34856190

[5] Lenders CM , McElrath TF , Scholl TO . Nutrition in adolescent pregnancy. Curr Opin Pediatr 2000;12:291–6. 10.1097/00008480-200006000-00021 10836168

[6] Jiwani SS , Gatica-Domínguez G , Crochemore-Silva I , et al . Trends and inequalities in the nutritional status of adolescent girls and adult women in sub-Saharan Africa since 2000: a cross-sectional series study. BMJ Glob Health 2020;5:e002948. 10.1136/bmjgh-2020-002948 PMC7545504 33033052

[7] Mtongwa RH , Festo C , Elisaria E . A comparative analysis of determinants of low birth weight and Stunting among under five children of adolescent and non-adolescent mothers using 2015/16 Tanzania demographic and health survey (TDHS). BMC Nutr 2021;7:64. 10.1186/s40795-021-00468-6 34732260 PMC8567641

[8] Nabugoomu J , Seruwagi GK , Corbett K , et al . Needs and barriers of teen mothers in rural Eastern Uganda: Stakeholders' perceptions regarding maternal/child nutrition and health. Int J Environ Res Public Health 2018;15:2776. 10.3390/ijerph15122776 30544550 PMC6314007

[9] Opiyo CO , Okeyo DO , Gumo S , et al . Power Dynamics as a determinant of access and utilisation of nutrition services by pregnant and lactating adolescent girls in Trans-Mara East sub-County. BMC Public Health 2020;20:537. 10.1186/s12889-020-08690-w 32306983 PMC7168838

[10] Jama NA , Wilford A , Haskins L , et al . Autonomy and infant feeding decision-making among teenage mothers in a rural and urban setting in Kwazulu-natal, South Africa. BMC Pregnancy Childbirth 2018;18:52. 10.1186/s12884-018-1675-7 29454323 PMC5816555

[11] Gyimah LA , Annan RA , Apprey C , et al . Dietary diversity and its correlates among pregnant adolescent girls in Ghana. PLoS One 2021;16:e0247979. 10.1371/journal.pone.0247979 33684165 PMC7939348

[12] Okeyo DO , Gumo S , Munde EO , et al . Nutritional service needs of pregnant and lactating adolescent girls in Trans-Mara East sub-County, Narok County: focus on access and utilisation of nutritional advice and services. BMC Pregnancy Childbirth 2019;19:229. 10.1186/s12884-019-2391-7 31277585 PMC6612069

[13] Fulpagare PH , Saraswat A , Dinachandra K , et al . Antenatal care service utilisation among adolescent pregnant women–evidence from Swabhimaan programme in India. Front Public Health 2019;7:369. 10.3389/fpubh.2019.00369 31921737 PMC6927275

[14] Self A , Chipokosa S , Misomali A , et al . Youth Accessing reproductive health services in Malawi: drivers, barriers, and suggestions from the perspectives of youth and parents. Reprod Health 2018;15:108. 10.1186/s12978-018-0549-9 29921282 PMC6008927

[15] Kenya Go . Kilifi early warning bulletin. 2021.

[16] Cheruiyot SJ , Kimanthi M , Shabani JS , et al . Climate change poses a threat to nutrition and food security in Kilifi County, Kenya. Afr J Prim Health Care Fam Med 2022;14:e1–4. 10.4102/phcfm.v14i1.3718 PMC9634675 36331200

[17] Kenya Demographic Health Survey 2022, Available: https://dhsprogram.com/pubs/pdf/PR143/PR143.pdf

[18] KNBS and ICF . Kenya Demographic and Health Survey 2022. Key Indicators Report. Nairobi, Kenya, and Rockville, Maryland, USA: KNBS and ICF, 2023.

[19] Kilifi County SMART Survey Report November, 2016. Available: http://www.nutritionhealth.or.ke/wp-content/uploads/SMART%20Survey%20Reports/Kilifi%20County%20SMART%20Survey%20Report%20November2016.pdf

[20] Contento IR . Nutrition education: linking research, theory, and practice. 2007. 18296331

[21] Government of Kenya . n.d. National climate change action plan (Kenya): 2018–2022.

[22] Tong A , Sainsbury P , Craig J . Consolidated criteria for reporting qualitative research (COREQ): a 32-item checklist for interviews and focus groups. Int J Qual Health Care 2007;19:349–57. 10.1093/intqhc/mzm042 17872937

[23] Kiger ME , Varpio L . Thematic analysis of qualitative data: AMEE guide No.131. Med Teach 2020;42:846–54. 10.1080/0142159X.2020.1755030 32356468

[24] Ngcamu BS , Chari F . Drought influences on food insecurity in Africa: Asystematic literature review. IJERPH 2020;17:5897. 10.3390/ijerph17165897 32823825 PMC7460121

[25] Tirado MC , Crahay P , Mahy L , et al . Climate change and nutrition: creating a climate for nutrition security. Food Nutr Bull 2013;34:533–47. 10.1177/156482651303400415 24605700

[26] Bett B , Kiunga P , Gachohi J , et al . Effects of climate change on the occurrence and distribution of livestock diseases. Prev Vet Med 2017;137:119–29. 10.1016/j.prevetmed.2016.11.019 28040271

[27] Sheriff M , Mash R . Climate change and primary health care in Chakama, Kilifi County, Kenya. Afr j Prim Health Care Fam Med 2022;14. 10.4102/phcfm.v14i1.3670 PMC9575365 36226937

[28] Ziro JS , Kichamu-Wachira E , Ross H , et al . Adoption of climate resilient agricultural practices among the Giriama community in South East Kenya: implications for conceptual frameworks. Front Clim 2023;5:1032780. 10.3389/fclim.2023.1032780

[29] Palinkas LA , Wong M . Global climate change and mental health. Curr Opin Psychol 2020;32:12–6. 10.1016/j.copsyc.2019.06.023 31349129

[30] Macheka L , Mudiwa T , Chopera P , et al . Linking climate change adaptation strategies and nutrition outcomes: A conceptual framework. Food Nutr Bull 2022;43:201–12. 10.1177/03795721221078362 35196891

[31] Bryan E , Theis S , Choufani J , et al . Gender-sensitive, climate-smart Agriculture for improved nutrition in Africa south of the Sahara. Annual Trends and Outlook Report 2017.

[32] Grace K , Davenport F , Funk C , et al . Child malnutrition and climate in sub-Saharan Africa: an analysis of recent trends in Kenya. Applied Geography 2012;35:405–13. 10.1016/j.apgeog.2012.06.017

[33] Debela BL , Demmler KM , Rischke R , et al . Maternal nutrition knowledge and child nutritional outcomes in urban Kenya. Appetite 2017;116:518–26. 10.1016/j.appet.2017.05.042 28558957

[34] Burchi F . Child nutrition in Mozambique in 2003: the role of mother’s schooling and nutrition knowledge. Econ Hum Biol 2010;8:331–45. 10.1016/j.ehb.2010.05.010 20646971

[35] Appoh LY , Krekling S . Maternal nutritional knowledge and child nutritional status in the Volta region of Ghana. Matern Child Nutr 2005;1:100–10. 10.1111/j.1740-8709.2005.00016.x 16881885 PMC6860941

[36] World Bank Climate Risk Country Profile: Kenya, Available: https://climateknowledgeportal.worldbank.org/sites/default/files/2021-05/15724-WB_Kenya%20Country%20Profile-WEB.pdf

