# Supplementary material for: Reimagining nutrition education for pregnant adolescents in the face of climate change: a community approach
Source: BMJ Nutr Prev Health. 2024 Apr 24;7(1):151–9. doi: 10.1136/bmjnph-2023-000745 (PMC11221292; doi:10.1136/bmjnph-2023-000745)
Supplement: Supplementary data [file bmjnph-2023-000745supp001.pdf]

APPENDIX 1 Focus Group Discussion Question Guide Adolescents – English Version

March 2022

Introduction: discussion enquiring about challenges with nutrition and nutrition education services for adolescents/young mothers and how they could be improved.

1. What challenges have you encountered in making a balanced meal for yourself and your children?
2. What foods do you easily have access to?
3. Has anything changed in recent years with regards to access to food?
4. What is your source of information on (adolescent pregnancy) nutrition and infant feeding?
5. What formal instruction have you received with regards to nutrition in adolescent pregnancy and infant feeding and where, when was this?
6. What parts of education were most helpful? What do you feel you need more of?
7. What is your current knowledge on nutrition: what food groups do you know and how many of them do you think are needed daily?
8. Are services currently offered in line with what you would need?
9. What are your suggestions for improvement? What should happen to improve the current state of affairs?

November 2022

Summary given on what was discussed during focus group discussion in November 2022 and checking with participants if this is indeed what they had reported.

1. Have you met any new challenges in making a balanced meal for yourself and your children?
2. You mentioned prolongued droughts as a reasons for difficulty in accessing food, what foods do you easily have access to?
3. Has anything changed in recent years with regards to access to food?
4. What formal instruction have you received with regards to nutrition in adolescent pregnancy and infant feeding and where, when was this?
5. What parts were most helpful? What do you feel you need more of?
6. What is your source of information on adolescent pregnancy nutrition and infant feeding?
7. What is your current knowledge on nutrition: what food groups do you know and how

many of them do you think are needed daily?

8. Are services currently offered in line with what you would need?
9. What are your suggestions for improvement? What should happen to improve the current state of affairs?
10. You mentioned men should be more involved in nutrtion education. What are ways to involve men in nutrition education?
